# *In Vitro* Phase Separation Characterization of the *Arabidopsis thaliana* Glycine-Rich RNA-Binding Protein AtGRP2

**DOI:** 10.1021/acsomega.6c02622

**Published:** 2026-05-21

**Authors:** Giovanna S. Melo, Gilberto Sachetto-Martins, André L. S. Santos, Yraima Cordeiro, Anderson S. Pinheiro

**Affiliations:** † Graduate Program in Biochemistry (PPGBq), Institute of Chemistry, 28125 Federal University of Rio de Janeiro, Rio de Janeiro 21941-909, Brazil; ‡ Department of Genetics, Institute of Biology, Federal University of Rio de Janeiro, Rio de Janeiro 21941-902, Brazil; § Department of General Microbiology, Institute of Microbiology Paulo de Góes, Health Science Center, Federal University of Rio de Janeiro, Rio de Janeiro 21941-902, Brazil; ∥ Faculty of Pharmacy, Federal University of Rio de Janeiro, Rio de Janeiro 21941-902, Brazil

## Abstract

The *Arabidopsis thaliana* glycine-rich RNA-binding protein 2 (AtGRP2) belongs to the cold-shock domain (CSD) protein family and regulates flowering and abiotic stress responses. It comprises an N-terminal RNA recognition motif (RRM) and a C-terminal intrinsically disordered glycine-rich domain (GRD) implicated in phase separation (PS). Herein, we investigated the PS behavior of AtGRP2 *in vitro* and its modulation by RNA. PS of recombinant AtGRP2 was monitored by fluorescence spectroscopy and turbidity measurements. Under crowding conditions, the full-length protein formed spherical, micron-sized droplets with low fusion dynamics, indicating a viscoelastic state. These condensates were resistant to high salt but disrupted by 1,6-hexanediol, highlighting the role of hydrophobic interactions in PS. AtGRP2 condensation was disrupted in the presence of total *Arabidopsis* RNA or the Lin28-specific miRNA, a prelet-7g precursor, in a dose-dependent manner. Lin28 is a human counterpart to AtGRP2, sharing similar domains except for the GRD. Prelet-7g was found to partition into the condensates and directly interact with AtGRP2. Mutational analysis showed that the CSD alone was insufficient for PS, while the isolated C-terminal region formed solid aggregates. Deletion of the GRD had little impact on phase behavior; however, removal of the CCHC-type zinc finger or arginine residues completely abolished droplet formation, highlighting their critical role in mediating the multivalent interactions required for PS. These results demonstrate AtGRP2 undergoes PS driven by its disordered C-terminal region and modulated by RNA binding. This mechanism may contribute to its localization to membraneless organelles, including the nucleolus, supporting stress adaptation similarly to Lin28.

## Graphical abstract

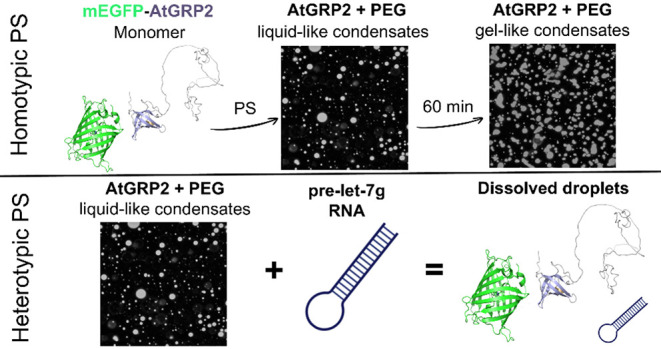

## Introduction

Phase separation (PS) is a physical phenomenon in which a supersaturated macromolecular solution spontaneously separates into two coexisting liquid phases: one dense and one dilute. Initially observed in polymers and proteins, like hemoglobin, PS is now recognized as a key mechanism for forming membraneless cellular structures called biomolecular condensates, which regulate biological activities.^1^ These condensates, composed of proteins, RNA, and other biomolecules, can transition between homogeneous mixtures and spatially organized phases.^1^ Multivalent interactions, mediated by folded domains or intrinsically disordered regions (IDRs), drive PS. IDRs, enriched in low-complexity domains (LCDs) and short linear interaction motifs (SLiMs), facilitate weak, reversible interactions (*e.g.*, π–π, cation-π) that enable dynamic condensate formation.^2–4^ Despite progress, research on PS in plants still focuses mainly on biological phenomena, with limited biophysical and structural insight into the condensed phase. Integrating molecular biology with biochemical, imaging, and computational approaches will be essential to clarify how phase-separating proteins function under both normal and stress conditions.^5^

RNA-binding proteins (RBPs) play a fundamental role in post-transcriptional gene regulation by participating in RNA splicing, stability, transport, translation, and degradation.^6^ They interact with *cis*-regulatory RNA elements and *trans*-acting factors, including other RBPs and noncoding RNAs.^7^ Structurally, RBPs display modular RNA-binding domains, such as the RNA recognition motif (RRM), K-homology (KH) domain, cold shock domain (CSD), zinc finger, sterile alpha motif (SAM), and PIWI–Argonaute–Zwille (PAZ) domain, which confer specificity for RNA targets.^8^ These proteins modulate alternative splicing,^9^ RNA editing,^10^ nuclear export,^11^ mRNA stability,^12^ and translation.^13^ RNA fate depends on the cellular repertoire of RBPs, which often assemble into ribonucleoprotein particles (RNPs) together with other regulatory factors to ensure precise control of gene expression.^14^

Among the diverse RBP families, glycine-rich proteins (GRPs) are particularly noteworthy because of their structural and functional versatility. GRPs are characterized by glycine repeats and play essential roles in plant stress responses.^15^ They participate in processes such as cell wall formation, regulation of flowering, pathogen defense, and interactions with protein kinases.^16^ Different GRP classes show distinct expression patterns in response to hormones, developmental stages, and environmental stimuli.^15^ GRPs are classified into five classes (I–V) based on their domains and glycine motifs. Class IV, subdivided into IVa–IVd, comprises RNA-binding proteins. Subclass IVa contains an RRM domain and a glycine-rich tail; IVb features RRM and CCHC zinc finger domains; IVc includes a cold shock domain (CSD) and multiple zinc fingers (ZnFs); and IVd contains two RRM domains.^15,16^ The most common structural domains in Class IV GRPs are RRMs, which bind RNA and participate in splicing and poly­(A) tail processing.^17,18^ The CSD binds DNA and RNA and functions as an RNA chaperone during the cold response, often in association with glycine-rich regions.^19^ Glycine-rich RNA-binding proteins (GR-RBPs), particularly Class IV members, are involved in both developmental processes (*e.g.*, germination and growth) and stress responses (*e.g.*, cold, salinity, drought, and biotic stress).^20^

A key example of a GRP is AtGRP2 (AtCSP2), a 19 kDa glycine-rich protein (∼40% glycine) that features an N-terminal cold shock domain (CSD) and a C-terminal glycine-rich region containing two CCHC-type zinc finger domains.^21^ This domain architecture classifies AtGRP2 as a multifunctional Class IVc RNA-binding protein. AtGRP2 is primarily localized in the nucleus, with strong accumulation in the nucleolus, but it is also found in the cytoplasm, as demonstrated using fluorescently labeled AtGRP2 ^22^ and nucleolar markers.^23^ AtGRP2 expression is highest in tissues with proliferative activity and is induced by abiotic stress, particularly cold stress. AtGRP2 functions as an RNA chaperone and is essential for floral development, seed formation, and regulation of flowering time.^22^ Gene silencing results in flowers with fewer stamens and abnormal seed development,^22^ whereas overexpression causes delayed flowering and aberrant silique development.^24^ Notably, AtGRP2 negatively regulates freezing and salt tolerance. Loss-of-function mutants (atcsp2–3 and atcsp4–1) exhibit enhanced stress resistance, whereas overexpressing lines show increased sensitivity.^23^ AtGRP2 acts as a negative regulator of the CBF pathway, which controls cold-responsive (COR) gene expression. Reduced AtGRP2 levels increase CBF and COR gene expression and enhance tolerance, whereas AtGRP2 overexpression suppresses this pathway.^24^

Given the growing recognition of the central role of PS in cellular organization and plant stress responses,^5^ we aimed to elucidate the molecular mechanisms that regulate the condensation behavior of the *Arabidopsis thaliana* protein AtGRP2 by integrating physicochemical and biological approaches. Building on previous findings showing that the CSD of AtGRP2 alternates between conformational states and interacts specifically with oligonucleotides through π-stacking interactions,^25^ we investigate how AtGRP2 contributes to the dynamic formation and viscoelastic properties of biomolecular condensates.

To this end, we performed a comprehensive *in vitro* investigation of the molecular mechanisms underlying AtGRP2 phase separation. We examined the electrostatic interactions that drive PS and assessed the modulatory role of RNA in condensate stability and function. In addition, we evaluated the response of AtGRP2 to physiologically relevant environmental parameters, including pH, ionic strength, and molecular crowding. Together, these experiments provide a systematic *in vitro* framework to dissect the physicochemical determinants governing AtGRP2 PS. To our knowledge, this work provides one of the most comprehensive data sets describing the structural and functional principles of PS in plant RNA-binding proteins. These results help address key gaps in the field and provide a basis for future strategies to modulate gene expression and improve plant resilience to environmental stress through proteins with tunable PS properties.

## Results

### Propensity for Disorder and PS of AtGRP2

PS is frequently mediated by intrinsically disordered proteins (IDPs) or intrinsically disordered regions (IDRs) that lack a defined three-dimensional structure.^26^ Many IDPs/IDRs contain low-complexity regions characterized by amino acid sequence repeats.^26^ In the case of AtGRP2, its glycine-rich C-terminal tail exhibits low sequence complexity, making it a strong candidate for PS. Here, we employed multiple bioinformatic algorithms to predict structural disorder and the propensity for condensate formation from AtGRP2′s primary structure.

Using PONDR predictors, we analyzed the disorder propensity of AtGRP2. While only the VLXT predictor suggested that the N-terminal CSD (residues 1–90) is fully ordered, the other predictors indicated moderate disorder (score ∼ 0.5) in this region (Figure 1A). These predictions are consistent with experimental data showing that the CSD of AtGRP2 exists in a conformational equilibrium between folded and unfolded states.^25^ The C-terminal glycine-rich region showed high disorder scores, with localized ordering only at the zinc finger domains (Figure 1A), supporting our hypothesis of a folded N-terminal domain coupled to a flexible, disordered C-terminal tail. We further characterized the disorder of AtGRP2 using charge–hydropathy analysis. Disordered proteins typically exhibit a higher content of charged amino acids and lower hydrophobicity than ordered proteins.^27^ AtGRP2 plotted near the order–disorder boundary (Figure 1B), suggesting either a predominantly disordered structure or a protein with alternating ordered and disordered regions. Residue-by-residue analysis revealed alternating charge patterning (characteristic of weak polyampholytes) and low mean hydrophobicity (∼0.5), particularly in the C-terminal tail, consistent with IDP-like features (Figure 1C,D).^28–30^ PScore analysis (prediction of π–π contacts) yielded a score of 9.07, with the highest values in the glycine-rich C-terminal region (Figure 1E). catGRANULE (protein–RNA granule prediction)^31^ indicated that the glycine-rich regions of AtGRP2 have a higher granule-forming propensity than FUS, a canonical model protein for PS. This analysis was benchmarked against control proteins known to undergo or not undergo PS, namely AtGRP7 (UniProt ID Q03250), RBM3 (P98179), FUS (P06748), Tau (P10636), bovine serum albumin (BSA; P02768), and Smac (Q9NR28) (Figure 1F). PLAAC (prion-like domain identification) detected strong prion-like signatures in residues 83–122 and 151–180 (Figure 1G), suggesting the potential for amyloid aggregation under specific conditions.^32,33^ The computational analyses strongly suggest that AtGRP2 undergoes PS mediated by its disordered C-terminal tail and may also display prion-like aggregation behavior. The remarkably high catGRANULE score (exceeding that of FUS) provides compelling evidence for its PS propensity.^34,35^ However, experimental validation through condensation assays and cellular models remains essential to fully characterize this phenomenon.^34–36^ Taken together, our *in silico* analyses support a model in which the glycine-rich C-terminal region of AtGRP2 primarily acts as a spacer that modulates solubility, conformational flexibility, and effective interaction valence, whereas localized aromatic and prion-like segments may function as stickers. In this framework, PS is not driven by a single dominant interaction but instead emerges from the collective behavior of disordered, weakly interacting motifs, consistent with contemporary stickers-and-spacers models.

**Figure 1:**
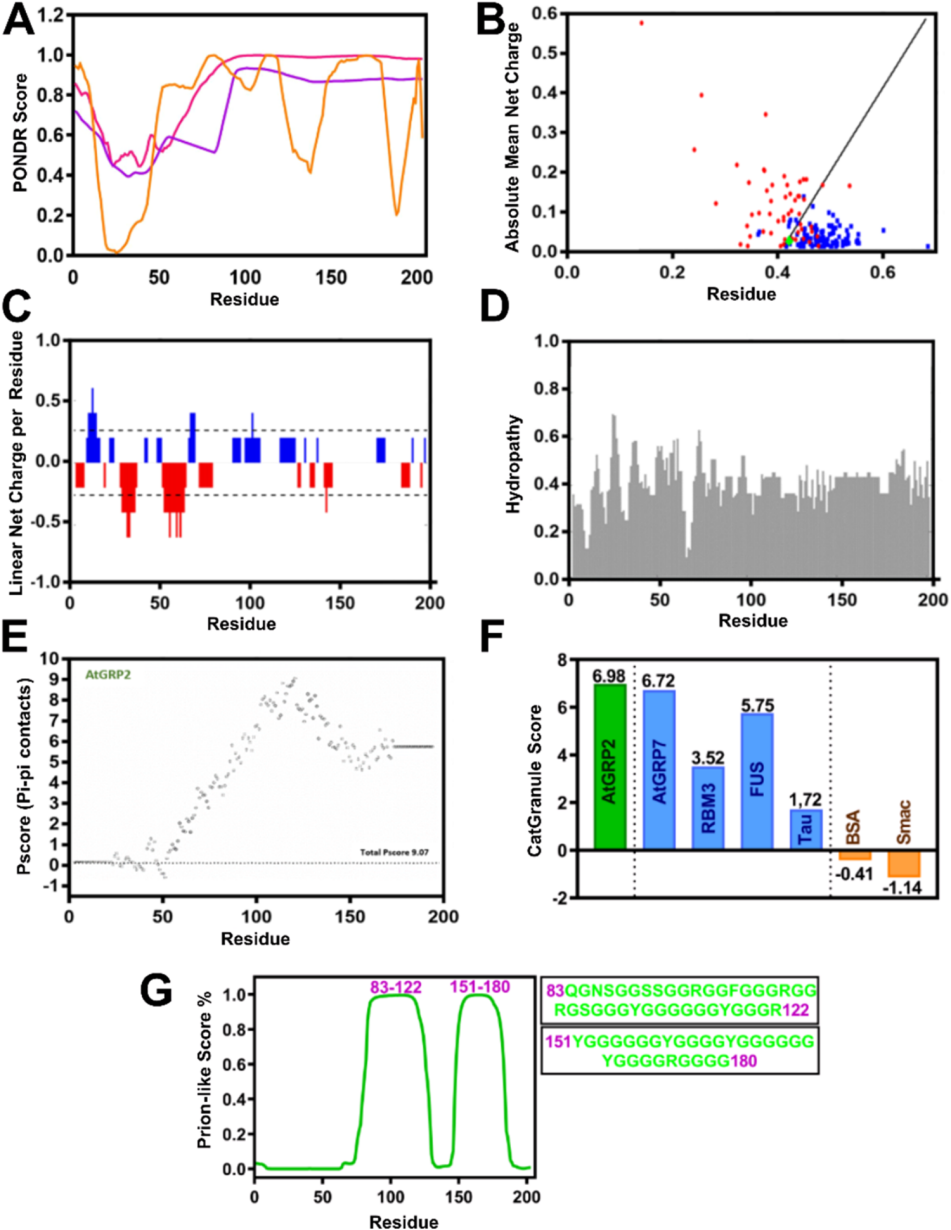
Disorder propensity and LLPS analysis of AtGRP2. (A) Residue-by-residue disorder prediction for AtGRP2 was performed using PONDR (Predictor of Natural Disordered Regions) algorithms (VLXT, orange; VL3, purple; VLS2, pink). (B) Charge–hydropathy (CH) plot for AtGRP2 generated by PONDR. Red dots represent disordered proteins reported in the literature, blue dots represent ordered proteins, and the green dot represents AtGRP2. (C) Net charge per residue plot for AtGRP2 generated by CIDER (Classification of Intrinsically Disordered Ensemble Regions) shows positively charged segments in blue and negatively charged segments in red. The alternating charge pattern indicates that AtGRP2 is a weak polyampholyte, a feature common in proteins that undergo electrostatically driven phase separation. (D) Hydropathy plot along the AtGRP2 amino acid sequence, also generated by CIDER, shows the distribution of hydrophobicity. (E) PScore analysis predicts elevated π–π contact propensity in the glycine-rich C-terminal tail. (F) LLPS propensity based on the catGRANULE score, comparing AtGRP2 with control proteins known to undergo (or not undergo) PS. AtGRP2 is shown in green. Blue bars represent positive controls: AtGRP7 (UniProt ID Q03250), RBM3 (P98179), FUS (P06748), and Tau (P10636). Orange bars indicate negative controls: bovine serum albumin (BSA; P02768) and Smac (Q9NR28). Scores are indicated above each bar. (G) Prion-like score calculated using PLAAC (Prion-Like Amino Acid Composition).

### AtGRP2 Undergoes LLPS under Molecular Crowding Conditions

Using RNA-free AtGRP2, we conducted *in vitro* PS assays monitored by fluorescence microscopy of the monomeric enhanced green fluorescent protein (mEGFP) fusion protein. Microscopic analysis revealed the formation of spherical, micron-sized condensates (Figure 2A–C), indicative of PS. These structures were predominantly observed under molecular crowding conditions using 10% PEG 4000, which mimics the intracellular environment by increasing the excluded volume around protein molecules.^37,38^ Quantitative analysis demonstrated concentration-dependent increases in both condensate number and size, with PS becoming evident at ≥ 7.5% crowding agent and 10 μM AtGRP2 (Figure 2D). Light scattering measurements at 600 nm showed dose-dependent increases in turbidity with increasing PEG 4000 concentration at a fixed protein concentration (10 μM) (Figure 2E), confirming enhanced protein partitioning into the condensed phase. Experiments with alternative crowding agents (PEG 20,000 and Ficoll) yielded similar spherical condensates above 7.5% (Figure 2B,C) and comparable turbidity profiles (Figure 2E), indicating that AtGRP2 PS is independent of the specific crowding agent chemistry.^39,40^

**Figure 2:**
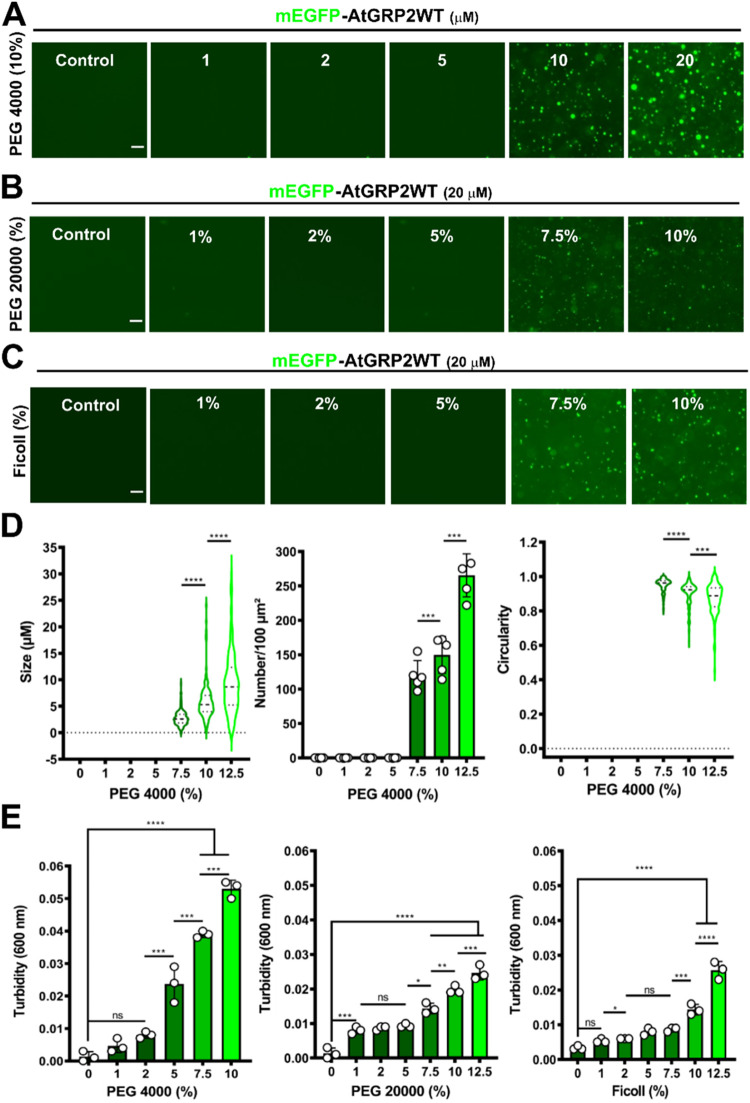
PS of AtGRP2 investigated by fluorescence microscopy and turbidity. (A–C) Representative fluorescence microscopy images of purified AtGRP2. Scale bars: 10 μm. Fluorescence microscopy showing PS of AtGRP2 under molecular crowding conditions (PEG 4000) (A). Fluorescence microscopy showing AtGRP2 (at 20 μM) condensate formation in the presence of increasing PEG 20000 (B) or Ficoll (C) concentrations. (D) Quantification of condensate size, number and circularity as a function of PEG 4000 concentrations with 20 μM AtGRP2. (E) Turbidity measurements at 600 nm showing dose-dependent increases in turbidity at a fixed AtGRP2 concentration (10 μM) with PEG 4000, PEG 20000, and Ficoll. Data in (D and E) are presented as mean ± SD; *n* = 3 independent experiments (same protein batch). Statistical analysis: one-way ANOVA with Tukey’s multiple-comparisons test; *p* < 0.05 (ns (*p* ≥ 0.05), *­(*p* < 0.05), **­(*p* < 0.01), ***­(*p* < 0.001), ****­(*p* < 0.0001)).

Biomolecular condensates can exhibit liquid, hydrogel, or solid states. True liquids display characteristic properties, including spherical morphology, the ability to fuse, and high molecular mobility.^41–43^ Fluorescence recovery after photobleaching (FRAP) analysis showed only ∼20% fluorescence recovery over 75 s (Figure 3A), indicating restricted molecular dynamics. Time-lapse microscopy showed that droplets did not fuse upon collision; instead, they adhered to each other, forming cluster-like assemblies (Figure 3B). Together, these findings suggest that AtGRP2 forms viscoelastic condensates with hydrogel-like properties rather than conventional liquid droplets.

**Figure 3:**
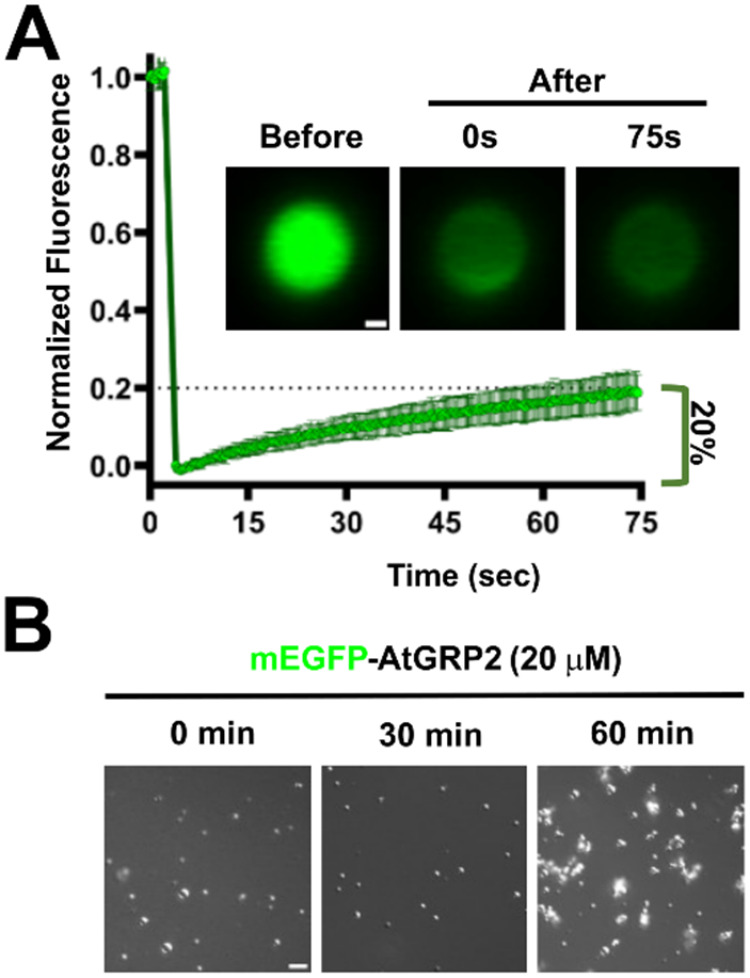
AtGRP2 forms gel-like condensates. (A) FRAP analysis of AtGRP2 at 20 μM showing partial fluorescence recovery over time. Scale bar: 1 μm. (B) Time-lapse microscopy showing gel-like behavior, with droplets adhering upon contact and forming cluster-like assemblies after 1 h of incubation. Scale bar: 1 μm.

PS condensates can undergo aging transitions, progressively evolving toward more stable states.^44,45^ Time-course microscopy showed that, whereas freshly formed AtGRP2 condensates displayed a spherical morphology, 1 h of incubation induced cluster formation without fusion events (Supporting Video 1). This adhesive behavior, together with the absence of liquid-like characteristics, suggests a rapid transition to a hydrogel state, a three-dimensional network with increased mechanical rigidity relative to liquid phases.^46^ These observations are consistent with evidence that proteins can exploit liquid-to-solid transitions for biological function^47^ and that hydrogel states often involve amyloid-like fibrils.^2,35^

### Cation-π and Hydrophobic Interactions Drive PS of AtGRP2

Experimental evidence suggests that a hierarchy of interactions governs protein PS, typically involving long-range electrostatic contacts and short-range cation-π interactions between aromatic residues (particularly tyrosine and phenylalanine) and positively charged residues (especially arginine).^48,49^ To characterize the interactions mediating AtGRP2 condensation, we examined the effects of NaCl, arginine, and 1,6-hexanediol on its PS Remarkably, increasing NaCl concentrations (up to 1 M) did not alter condensate number (Figure 4A) and only modestly affected condensate size above 300 mM, with turbidity measurements confirming this resistance (Figure 4B). These findings indicate that polar interactions play a minimal role in AtGRP2 PS. Bioinformatic analyses suggest that the Gly/Arg/Tyr-rich C-terminal region likely drives condensation through alternative mechanisms. Given this electrostatic resilience, we investigated cation-π interactions between arginine guanidinium groups and tyrosine aromatic rings. Arginine titration caused dose-dependent reductions in condensate number and turbidity, suggesting that free arginine competes with protein-bound arginine for interactions with tyrosine residues (Figure 4C,D).

**Figure 4:**
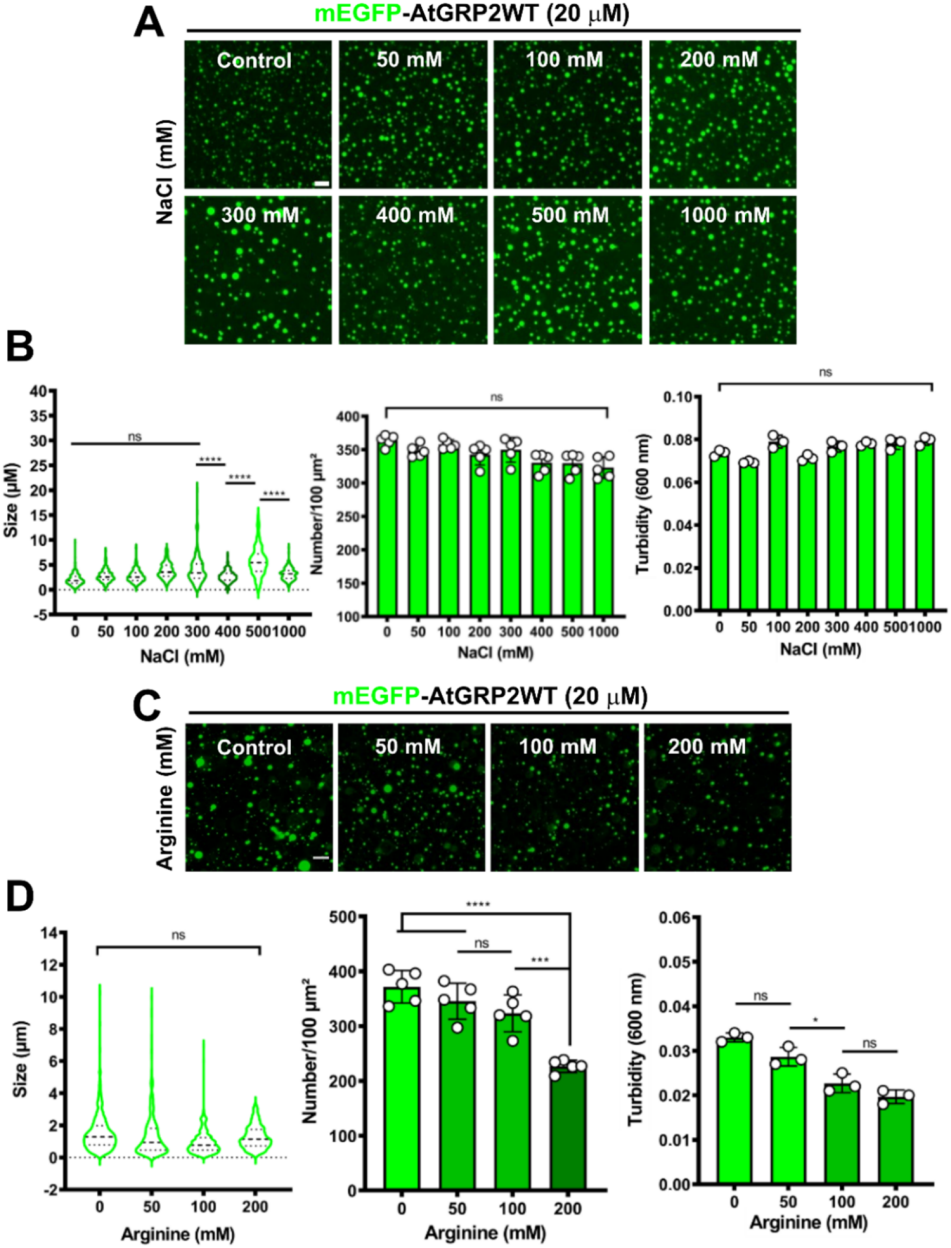
AtGRP2 condensates are sensitive to NaCl and arginine. (A) Fluorescence microscopy showing that 20 μM AtGRP2 condensates remain stable even at high NaCl concentrations (1 M). (B) Quantification of condensate number and size, together with turbidity measurements, indicating AtGRP2 condensate stability up to 1 M NaCl. (C) Fluorescence microscopy showing significant reduction in condensates at arginine concentrations >100 mM. Scale bar: 10 μm. (D) Quantification of condensate number and size, together with turbidity measurements, confirming the arginine-dependent reduction in condensate formation. Data in (B and D) are presented as mean ± SD; *n* = 3 independent experiments (same protein batch). Statistical analysis: one-way ANOVA with Tukey’s multiple-comparisons test; *p* < 0.05 (ns (*p* ≥ 0.05), *­(*p* < 0.05), **­(*p* < 0.01), ***­(*p* < 0.001), ****­(*p* < 0.0001)).

Notably, these cation-π interactions have both electrostatic and dispersion components, with parallel-stacked conformations being dispersion-dominated,^50^ which may explain their salt resistance. Treatment with 1,6-hexanediol, which disrupts weak hydrophobic interactions,^51^ significantly reduced both condensate number and turbidity (Figure 5A,B). This sensitivity, together with the Tyr-rich C-terminal sequence, suggests that π–π stacking between aromatic rings cooperates with cation−π interactions to drive PS. Basic pH (>7.5) prevented condensate formation, whereas acidic conditions (<5.0) induced amorphous solid aggregates (Figure 5C). Notably, AtGRP2 fused to maltose-binding protein (MBP–AtGRP2) used as a control construct with an alternative fusion partner, formed networked aggregates at pH 4.5 (50 μM), indicating concentration-dependent aggregation (Figure 5D). Turbidity measurements confirmed these transitions: the decreased signal at basic pH reflected dissolution, whereas acidic conditions showed increased scattering due to solid aggregation (Figure 5E).

**Figure 5:**
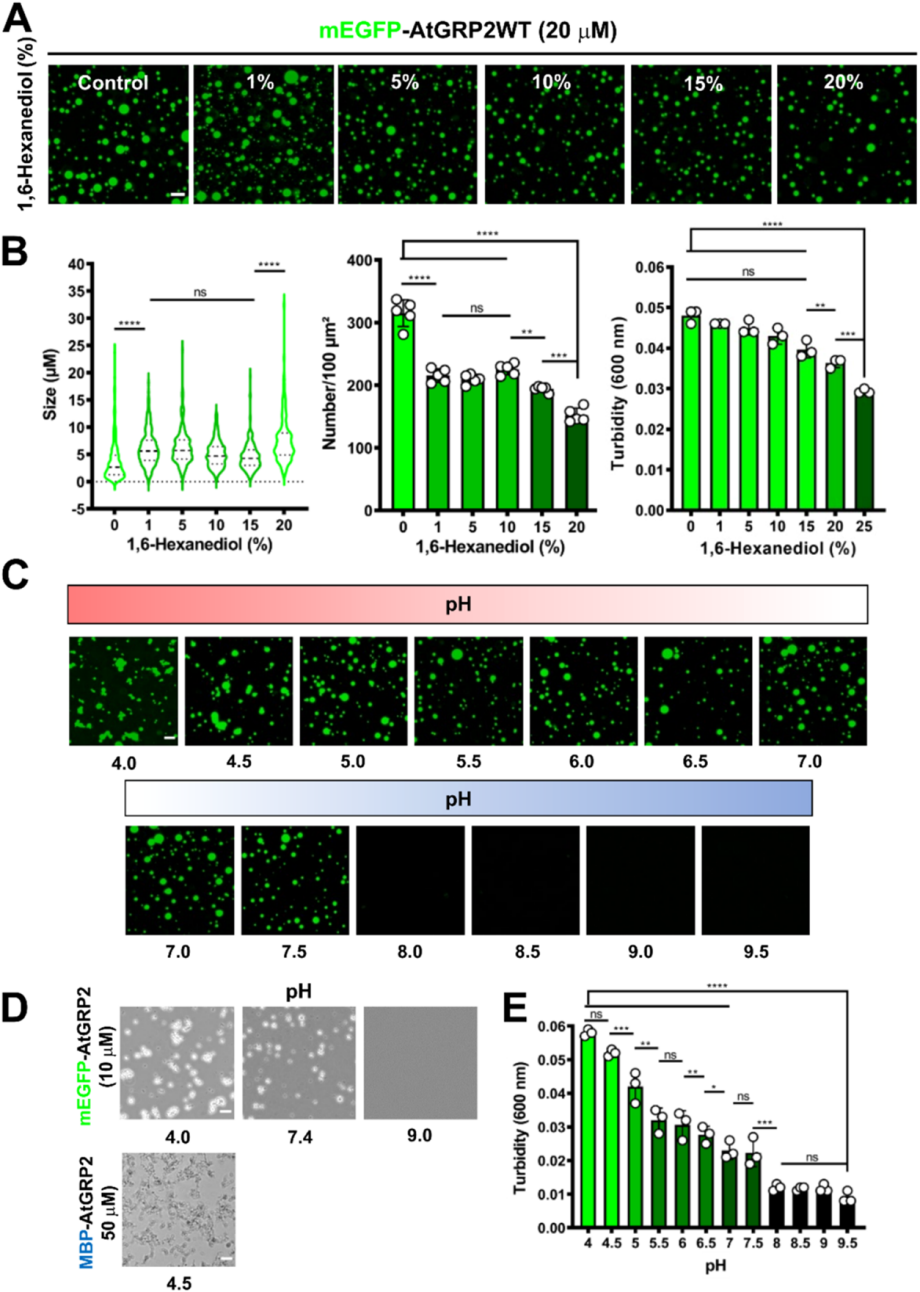
AtGRP2 condensates are sensitive to 1,6-hexanediol and pH. (A) Fluorescence microscopy showing decreased condensate formation upon treatment with 1,6-hexanediol. (B) Quantification of condensate size and number, together with turbidity measurements at 600 nm, confirming the reduction in phase separation observed in (A). (C) Fluorescence microscopy showing the absence of AtGRP2 condensates (at 10 μM) at pH > 7.5. Scale bar: 10 μm. (D) Phase-contrast microscopy indicates that acidic pH induces a liquid-to-solid transition in MBP-AtGRP2 and GFP-AtGRP2 condensates. (E) Turbidity measurements at 600 nm shows condensate dissolution at pH > 7.5 and increased scattering under acidic conditions, consistent with solid aggregation. Data in (B and E) are presented as mean ± SD; *n* = 3 independent experiments (same protein batch). Statistical analysis: one-way ANOVA with Tukey’s multiple-comparisons test; *p* < 0.05 (ns (*p* ≥ 0.05), *­(*p* < 0.05), **­(*p* < 0.01), ***­(*p* < 0.001), ****­(*p* < 0.0001)).

### RNA Maintains AtGRP2 in a Diffuse State by Disrupting Condensate Formation

Previous studies have established that RNA can bidirectionally modulate PS, either inducing PS or dissolving preformed condensates depending on stoichiometry.^52^ To investigate this phenomenon for AtGRP2, we first examined the effect of total *Arabidopsis* RNA extracts (containing ribosomal, messenger, and tRNAs) on preformed condensates. Dose–response experiments revealed that increasing RNA concentrations (up to 100 ng/μL) progressively reduced both condensate size and number, with complete dissolution observed at higher concentrations (Figure 6A). Turbidity measurements confirmed this dose-dependent disassembly (Figure 6B), suggesting that RNA competes with the homotypic protein–protein interactions that drive AtGRP2 PS. In addition, the ability of RNA to permeate and dissolve condensates supports a viscoelastic, liquid-like state rather than solid or hydrogel-like properties. Surprisingly, subsequent RNase A treatment (0.2 mg/mL) of RNA-dissolved samples failed to restore condensates (Figure 6C), prompting us to investigate the effects of free nucleotides. Titration experiments demonstrated that dNTPs (dATP, dCTP, dGTP, and dTTP) dose-dependently suppressed condensate formation, with equimolar concentrations (relative to the protein) causing complete dissolution (Figure 6D). In RNase A assays, dNTPs were included to assess whether residual RNA fragments generated during digestion could contribute to this effect; however, incomplete RNA degradation or potential RNase-induced effects on the protein cannot be excluded. These results suggest that RNA degradation products may maintain AtGRP2 in a soluble state through nucleotide-protein interactions, a conclusion further supported by EMSA data (Supporting Figure 1).

**Figure 6:**
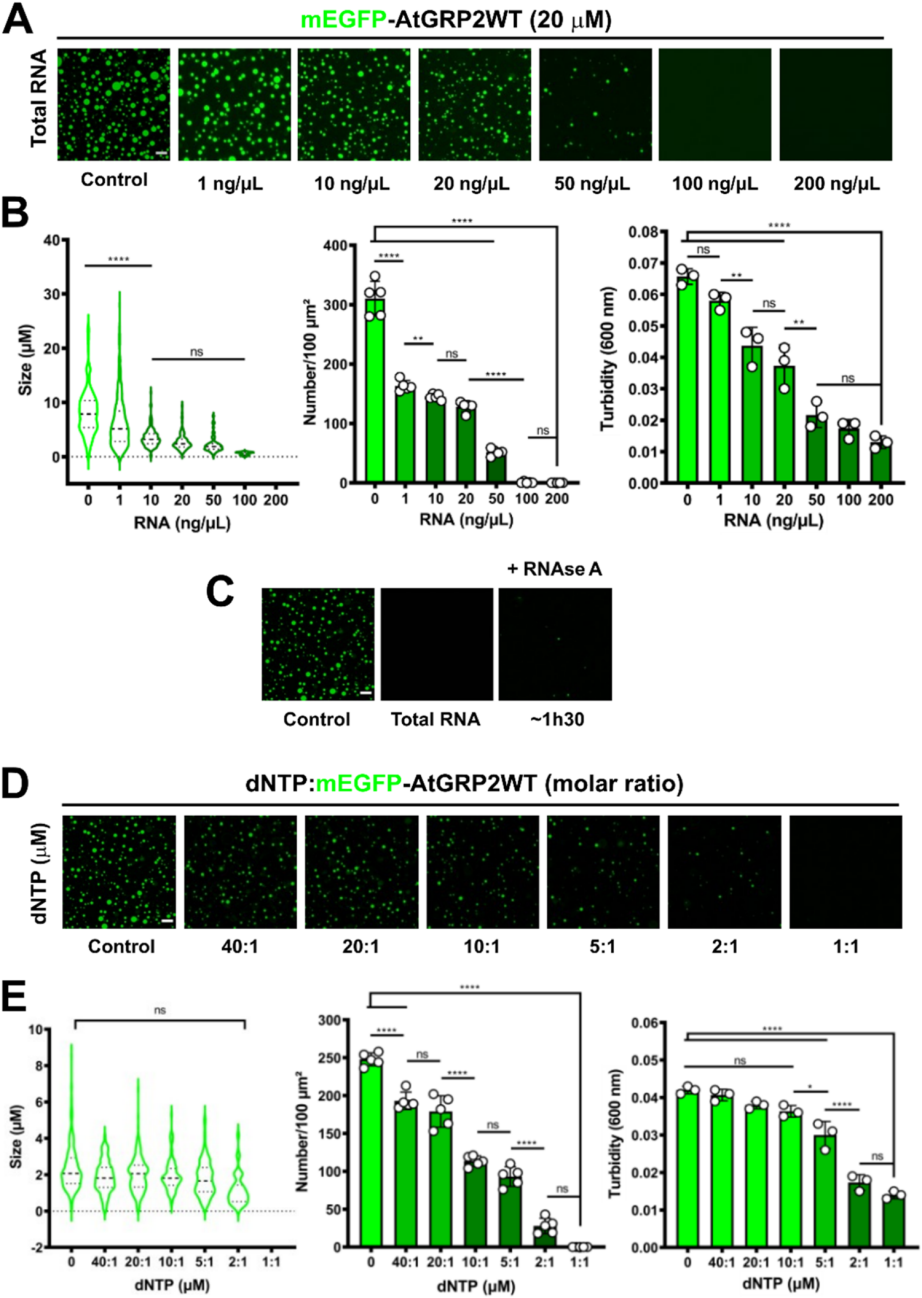
*Arabidopsis* total RNA dissolves AtGRP2 condensates. (A) Fluorescence microscopy showing reduced condensate formation upon addition of total *Arabidopsis* RNA extract. (B) Quantification of condensate size and number, together with turbidity measurements at 600 nm confirming RNA-dependent dissolution. (C) Fluorescence microscopy showing fewer condensates in the presence of total RNA (at 200 ng/ μL) or after treatment with RNase A (0.2 mg/mL). (D) Fluorescence microscopy of the dNTP:protein titration, showing the absence of condensates at high free-nucleotide concentrations (>10:1 dNTP:protein molar ratio). Scale bar: 10 μm. (E) Quantification of condensate size and number, together with turbidity measurements, confirming dNTP-dependent inhibition of condensate formation. Data in (B and E) are presented as mean ± SD; *n* = 3 independent experiments (same protein batch). Significance levels: ns (*p* ≥ 0.05), *­(*p* < 0.05), **­(*p* < 0.01), ***­(*p* < 0.001), ****­(*p* < 0.0001).

Pairwise sequence alignment between AtGRP2/AtCSP2 (At4g38680) and human Lin28A (UniProt Q9H9Z2), performed using Clustal Omega (global alignment, default parameters), revealed moderate sequence similarity (∼30–33%) (data not shown), consistent with their evolutionary distance. Despite the low global identity, both proteins share conserved architectural features, including an N-terminal cold shock domain, C-terminal CCHC zinc-binding motifs, and intrinsically disordered regions implicated in RNA binding and RNA-mediated phase behavior (Figure 7B). Given these structural similarities and the fact that the cognate RNA targets of AtGRP2 remain unknown, we investigated whether AtGRP2 can recognize Lin28′s well-characterized RNA ligand, prelet-7g miRNA, which regulates the transition from proliferative/immature to differentiated/reproductive states in animals. Although the functional RNA targets of AtGRP2 in *Arabidopsis* have yet to be identified, this parallel with Lin28 provides a conceptual framework suggesting that AtGRP2 may interact with plant miRNAs involved in developmental phase transitions, such as miR156 and miR172.

**Figure 7:**
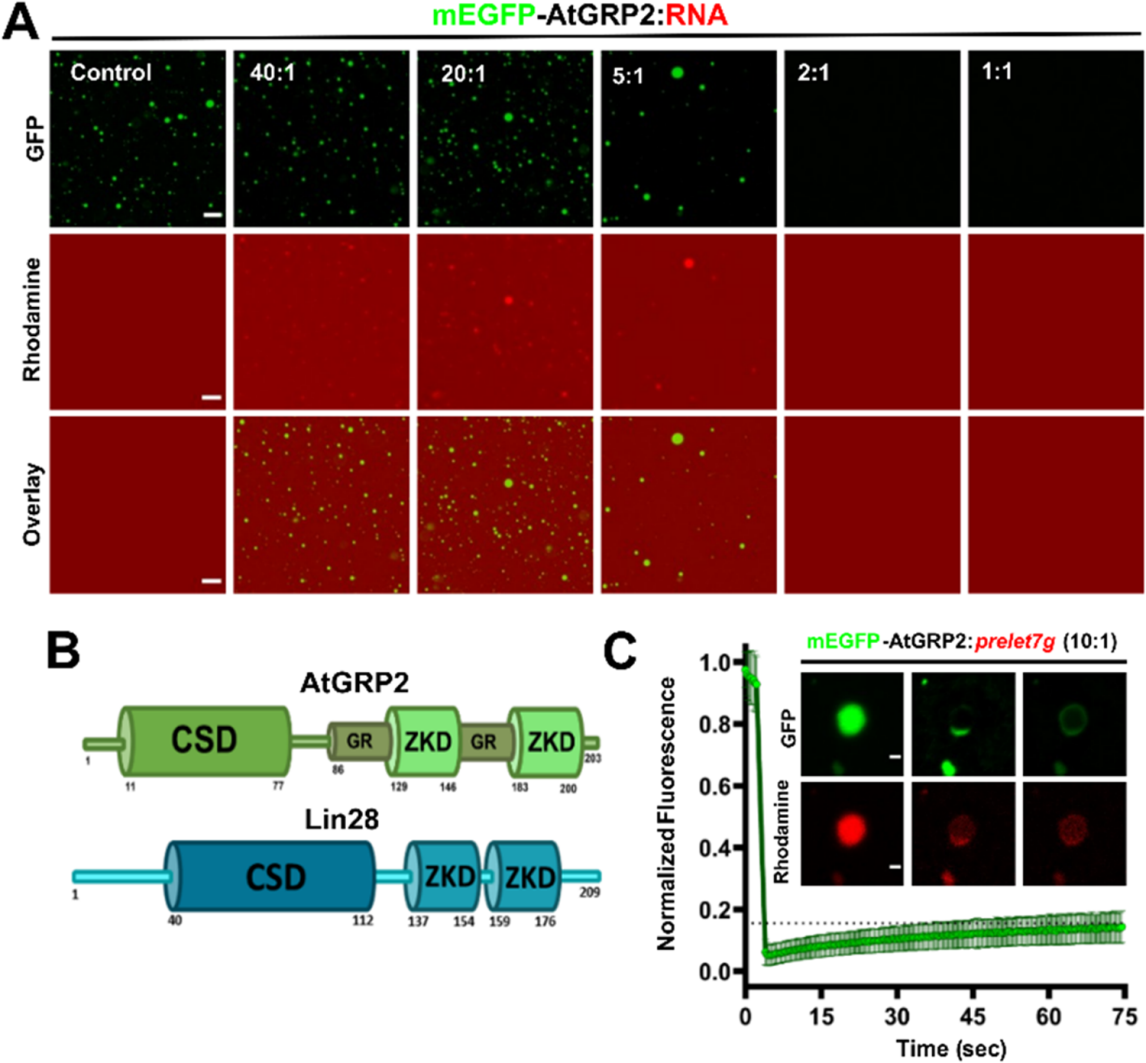
A Lin28 RNA ligand permeates and dissolves AtGRP2 condensates. (A) Fluorescence microscopy shows that rhodamine-labeled prelet-7g RNA penetrates AtGRP2 condensates (at 20 μM) and promotes their dissolution. Increasing protein:RNA molar ratios (1:1 to 40:1) were tested, as indicated, demonstrating RNA-dependent condensate disassembly. (B) Schematic domain architecture of Lin28A and AtGRP2, highlighting shared features (CSD and ZKD). (C) FRAP analysis of rhodamine-labeled RNA showing limited fluorescence recovery within AtGRP2 condensates. Scale bars: 1 μm.

Fluorescence microscopy showed that the rhodamine-labeled prelet-7g oligonucleotide (30 nt) permeated AtGRP2 condensates (Figure 7A), with complete dissolution occurring at a 2:1 protein:RNA stoichiometric ratio. FRAP analysis of the rhodamine-labeled RNA showed ∼20% fluorescence recovery (Figure 7C), matching the protein dynamics and indicating that RNA incorporation does not alter condensate material properties.

### The C-Terminal Tail Drives AtGRP2 PS

In this set of experiments, we generated a series of AtGRP2 mutant constructs to identify which domain(s) are responsible for its PS behavior. We analyzed six constructs: (i) the N-terminal region only, (ii) the C-terminal tail only, (iii) a construct lacking the zinc finger domains, (iv) a construct lacking glycine-rich regions, (v) a construct lacking tyrosine residues, and (vi) a construct lacking arginine residues (Supporting Table 1). The N-terminal-only construct (Supporting Figure 2A) did not form condensates, even at high protein concentrations (up to 1 mM), indicating that this region alone is insufficient to drive phase separation. Deletion of the zinc finger domains also abolished condensate formation (Supporting Figure 2B), raising the possibility that these domains promote the structural organization required for condensation. The arginine-deleted construct likewise did not undergo PS (Supporting Figure 2C), suggesting that Arg-mediated interactions are essential for condensation, likely through cation−π interactions and/or interactions with RNA or other biomolecules. The glycine-rich region–deleted construct retained phase-separation capability, but only at high protein concentrations (50 μM) and in the presence of 10% PEG as a crowding agent (Supporting Figure 2D). In contrast, the tyrosine-deleted construct (AtGRP2ΔTyr) underwent condensation at lower protein concentrations (≥2 μM) in the presence of 10% PEG. Even at lower PEG concentrations (1%), PS of AtGRP2ΔTyr was detected by turbidity at 20 μM, suggesting a stronger intrinsic propensity for PS (Supporting Figure 3A). The construct containing only the C-terminal tail (AtGRP2ΔNterminal) formed condensates at concentrations as low as 10 μM, although it displayed branched structures rather than spherical droplets (Supporting Figure 3B,C). These results suggest that the C-terminal domain is sufficient to drive PS, whereas the N-terminal region may act as a solubilizing element that contributes to the spherical morphology of the condensates.

All AtGRP2 constructs were expressed and purified following the same procedure established for the full-length protein. A representative SDS–PAGE analysis of the purified full-length AtGRP2 is shown (Supporting Figure 4). The full-length protein and the N-terminal deletion construct exhibited a saturation concentration (*C*_Sat_) of approximately 10 μM (Figure 8A,B). The construct lacking the glycine-rich region (AtGRP2ΔGly) showed an increased *C*_Sat_ (∼20 μM), indicating a lower propensity for PS (Figure 8C). In contrast, the tyrosine-deleted construct (AtGRP2ΔTyr) displayed a reduced *C*_Sat_ (∼5 μM), suggesting enhanced condensation at lower protein concentrations (Figure 8D). Conversely, constructs lacking the zinc finger domains, arginine residues, or the C-terminal region showed no detectable PS under the conditions tested, precluding determination of *C*_Sat_ (Figure 8E–G). These *C*_Sat_ values are consistent with the fluorescence microscopy data and quantitative analyses, reinforcing the robustness of our findings and highlighting the importance of these structural regions in modulating AtGRP2 phase behavior.

**Figure 8:**
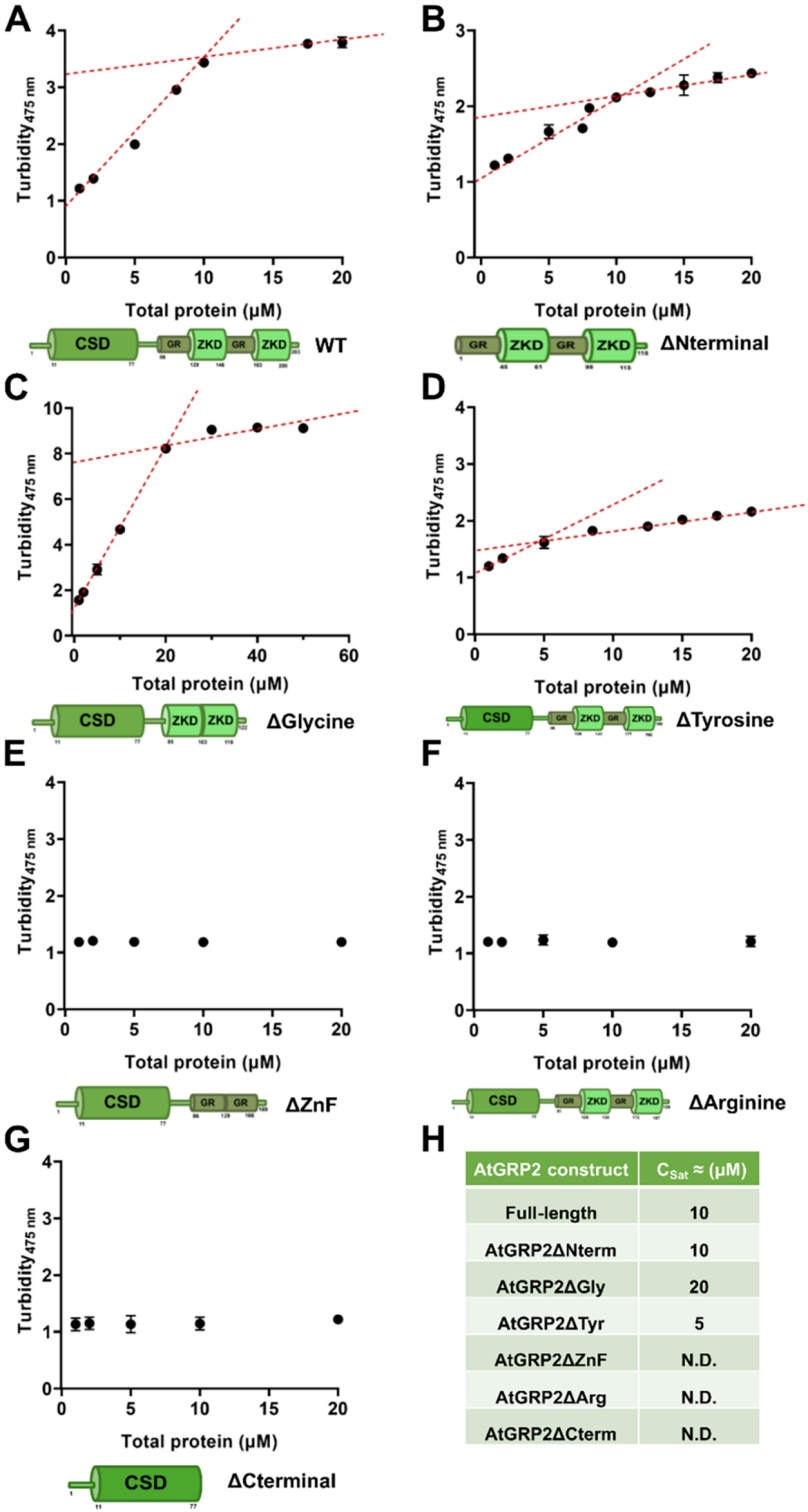
Saturation concentrations (*C*_Sat_) and schematic domain architecture of AtGRP2 constructs. (A) Full-length AtGRP2. (B) N-terminal deletion construct. (C) Construct lacking the glycine-rich region. (D) Tyrosine-deleted construct. (E) Zinc finger–deleted construct, (F) Arginine-deleted construct, no detectable phase separation under the conditions tested (no *C*_Sat_). (G) C-terminal deletion construct, no detectable phase separation under the conditions tested (no *C*_Sat_). (H) Corresponding saturation concentration (*C*_Sat_) determined from *in vitro* phase-separation assays. N.D., no detectable phase separation under the conditions tested (no *C*_Sat_).

## Discussion

In the present study, we generated a series of AtGRP2 variants with targeted mutations and altered domain compositions to identify the regions that are critical for condensate formation *via* LLPS and to assess how these regions influence RNA-binding properties. Our approach was intentionally designed as a comprehensive *in vitro* analysis, enabling the mechanistic dissection of intrinsic phase separation behavior and RNA-binding features in a controlled biochemical framework. The intracellular environment is highly crowded, with biomolecular concentrations ranging from 80 to 400 mg/mL.^50,51^ Molecular crowding is believed to play a pivotal role in regulating PS and promoting the formation of cellular condensates by increasing excluded-volume effects, thereby favoring compaction of intrinsically disordered regions in proteins.^52,53^ *In vitro* crowding agents, such as poly­(ethylene glycol) (PEG), mimic these effects by promoting biomolecular dehydration without partitioning into the condensed phase.^40^ Our results show that AtGRP2 undergoes PS under molecular crowding conditions, suggesting that this protein can self-organize into biomolecular compartments in the intracellular milieu. Notably, AtGRP2 formed condensates regardless of the crowding agent used, indicating that condensation is driven primarily by crowding as a physical effect rather than by specific interactions between the agent and the protein.^39^ Together, these results highlight the importance of molecular crowding in AtGRP2 condensation and support the relevance of our findings for understanding condensate formation in cells.

FRAP analysis revealed low fluorescence recovery for AtGRP2 in the condensed phase, indicating reduced protein mobility within the condensates and limited exchange with the surrounding dilute phase. Several factors may contribute to this low recovery. First, condensate formation leads to locally high protein concentrations that can hinder diffusion. Reduced mobility is consistent with the viscoelastic and relatively ordered nature of these assemblies and suggests increased stability and persistence of AtGRP2 condensates, likely due to a densely connected protein–protein interaction network. In addition, interactions with RNA may further reduce internal dynamics and promote material aging, thereby influencing condensate stability and dynamics.^54^ These findings may inform future strategies to modulate plant stress responses by targeting the physicochemical determinants of AtGRP2 condensate formation and stability.

Here, we investigated the formation of AtGRP2 condensates and observed that, after 1 h of incubation at room temperature, these assemblies organized into distinct clustered arrangements. This observation suggests that the condensates exhibit viscoelastic properties, combining features of a viscous liquid and an elastic solid. These results are consistent with previous studies reporting viscoelasticity in condensates formed by other proteins, including disease-associated FUS condensates.^55^ The formation of viscoelastic condensates is biologically relevant, as these material properties may influence intracellular function and dynamics. Viscoelasticity may also enable condensates to respond to mechanical stimuli and environmental fluctuations within the cell, thereby contributing to the regulation of cellular processes.

AtGRP2 exhibited remarkable resistance to NaCl treatment, which we used as an initial assay to probe the interactions underlying condensate formation *via* PS.^25^ Salt is known to screen electrostatic interactions and alter macromolecular solubility, potentially affecting condensate formation and integrity. However, the stability of AtGRP2 condensates in the presence of salt suggests that interactions beyond simple long-range electrostatics contribute to condensate formation and robustness. Hydrophobic interactions are critical for the stabilization and mechanical resilience of many protein condensates.^41^ Our findings suggest that such interactions, particularly π–π interactions involving Tyr residues in the C-terminal tail, together with cation−π interactions involving Arg residues, are key driving forces for AtGRP2 condensation.^3^

While polar electrostatic interactions do not appear to be the dominant forces driving AtGRP2 LLPS, cation−π interactions are likely to play a key role. In the C-terminal region, Arg residues may engage in cation−π interactions with Tyr residues *via* contacts between the Arg guanidinium group and the Tyr aromatic ring. Depending on the geometry, these interactions can include not only an electrostatic component but also a substantial dispersion contribution.^50^ Previous studies on RNA-binding proteins have highlighted the central role of Arg-mediated cation−π interactions with aromatic residues (*e.g.*, Trp) in mediating intermolecular contacts.^56^ These reports support our observations for AtGRP2 and reinforce the contribution of cation−π contacts to its phase behavior. Moreover, the viscoelasticity of protein condensates is thought to depend on networks of interactions among intrinsically disordered regions and with nucleic acids such as RNA. Interactions between cationic residues (*e.g.*, Arg and Lys) and aromatic residues (*e.g.*, Tyr and Phe) have been extensively studied and shown to contribute to protein organization and aggregation.^57^ Therefore, characterizing the nature and prevalence of cation−π interactions in AtGRP2 PS may help explain the viscoelastic properties of its condensates.

The interactions governing AtGRP2 PS likely arise from a combination of molecular forces, including π-π and cation-π interactions. The sensitivity of condensate formation to free arginine supports a central role for cation-π interactions in this process. Such interactions have been implicated in the stabilization of supramolecular assemblies, including protein condensates. In addition, sensitivity to 1,6-hexanediol suggests the involvement of hydrophobic contributions, including π-π contacts, in condensate formation. 1,6-Hexanediol is an amphiphilic compound known to disrupt weak hydrophobic interactions among protein molecules, thereby affecting condensate stability. Hydrophobic interactions are widely recognized as key drivers of protein condensation and aggregation.^58^ Regular spacing of aromatic residues within disordered regions can facilitate multivalent interactions and promote supramolecular assembly. Notably, the glycine-rich region of AtGRP2 contains multiple GGYGG repeats, providing regularly spaced Tyr residues that could similarly promote condensate formation through multivalency.^48,49^ Overall, these observations underscore the complexity of the interaction network that governs AtGRP2 condensate assembly and stabilization. Elucidating these interactions will be important for understanding the regulation of AtGRP2 phase behavior and its biological function.

Furthermore, it is essential to consider the influence of pH on condensate formation and dissolution, given the interactions that drive LLPS of AtGRP2. Under basic conditions (above pH 7.5), AtGRP2 condensates were fully dissolved. Given the role of π–π interactions and tyrosine residues in LLPS, the observed dissolution is plausibly due to the ionization of tyrosine’s phenolic hydroxyl group, leading to tyrosinate formation.^59^ Fluorescence spectroscopy assays targeting tyrosine residues as a function of pH, in both the dilute and condensed states, could help confirm this hypothesis. Conversely, under acidic conditions, condensates displayed an amorphous morphology, suggesting a transition to a solid-like phase. The melanosomal protein Pmel17 undergoes LLPS at physiological pH and transitions into solid structures at acidic pH (∼4.0).^60^ The formation of solid aggregates suggests the presence of strong and persistent intermolecular interactions. Although the solid structures formed by AtGRP2 at low pH are morphologically distinct from amyloid fibers, the presence of prion-like sequences within the glycine-rich C-terminal region of AtGRP2 raises the possibility of β-sheet-rich amyloid-like structures. Further experiments, such as secondary structure analysis and thioflavin T binding assays, are necessary to test this hypothesis. Our data indicate that AtGRP2 undergoes a pH-dependent liquid-to-solid phase transition, suggesting that this protein may act as an intracellular pH sensor, akin to other proteins.^61^ Given that intracellular acidification is associated with stress conditions such as anoxia in plants,^62^ this finding connects the phase behavior of AtGRP2 to stress response mechanisms.

Our results show that addition of total RNA extract from *A. thaliana* led to complete dissolution of AtGRP2 condensates. In our assays, condensates were first induced by poly­(ethylene glycol) and subsequently dissolved upon RNA addition, indicating that RNA can disrupt the interactions that sustain condensation. These findings also suggest that RNA can permeate the condensates, which display viscoelastic properties. Other RNA-binding proteins known to undergo phase separation, including EWSR1, TAF15, hnRNPA1, and TDP43, likewise form condensates that can be dissolved by RNA *in vitro*.^52^ Together, these results suggest that, in the intranuclear environment where RNA concentrations are high, AtGRP2 may be maintained in a soluble state through largely nonspecific interactions with diverse RNA species. This does not exclude the possibility that specific RNA sequences and/or structural elements may nucleate AtGRP2 condensation, as reported for FUS and NEAT1 RNA.^52^ Recent proteomic studies identified AtGRP2 in stress granules together with another glycine-rich RNA-binding plant protein, AtGRP7, suggesting that AtGRP2 can relocalize to the cytoplasm under stress conditions. Lower cytoplasmic RNA levels may then favor AtGRP2 condensation and promote its partitioning into stress granules.

AtGRP2 is structurally similar to Lin28. Both proteins comprise an N-terminal cold shock domain followed by two retroviral-type CCHC zinc finger domains. A key difference is the presence of glycine-rich regions, which are unique to AtGRP2. Lin28 localizes to the nucleolus during early embryogenesis, and its deletion results in defects in nucleolar assembly and activation of a p53-dependent stress pathway.^63,64^ The nucleolus is a spatially organized membraneless organelle formed by multiple immiscible liquid phases, each with a specific function.^65^ Similarly, AtGRP2 has been detected in the nucleolus and plays a role in *A. thaliana* embryonic development, raising the possibility that it contributes to nucleolar phase organization. In addition, we found that an RNA ligand recognized by Lin28 can permeate AtGRP2 condensates and promote their dissolution, suggesting that AtGRP2 can interact with this Lin28 ligand. Together, these observations support a model in which AtGRP2 partitions into the nucleolus during embryonic development *via* PS and may contribute to nucleolar integrity.^63,64^ Consistent with this hypothesis, previous interactome studies identified the nucleolar methyltransferase fibrillarin as a molecular partner of AtGRP2.^66^ Fibrillarin is a resident protein of the dense fibrillar component of the nucleolus.^65^ This finding opens new possibilities regarding the role of AtGRP2 phase separation in maintaining nucleolar organization, a process that may be important for embryonic development in *A. thaliana*.

Our results suggest that AtGRP2 phase separation is primarily driven by cation−π interactions between Arg and Tyr residues, with Gly residues acting as flexible spacers that modulate condensate dynamics. The glycine-rich C-terminal region of AtGRP2, which includes the zinc finger domains, is essential for PS and supports condensate formation at concentrations as low as 10 μM. Deletion of the N-terminal cold shock domain led to branched morphologies, consistent with a role for this region in promoting spherical, organized condensates. Deletion of Arg residues abolished phase separation, underscoring their critical role as “stickers” in cation−π interactions. Interestingly, removal of Tyr residues enhanced PS propensity, suggesting that, in this context, Tyr may act as a negative regulator of phase separation.^48^ Gly residues, due to their small side chain, confer conformational flexibility, which can facilitate condensate dynamics; consistent with this, deletion of glycine-rich segments increased the PS threshold concentration, supporting their role as “spacers”. Overall, these observations align with the stickers-and-spacers framework, in which specific residues drive interactions (stickers) while others modulate material properties (spacers). Studies on related proteins, such as FUS, similarly highlight the importance of Arg–Tyr cation−π interactions in PS.^67^ In summary, AtGRP2 PS is driven by cation−π interactions between Arg and Tyr residues in the C-terminal region, with Gly residues acting as flexible spacers. The N-terminal region influences condensate morphology by promoting spherical organization.

## Conclusion

Our *in vitro* data, together with the structural features and PS propensity of AtGRP2, suggest that this protein may participate in dynamic regulatory processes such as stress granule formation, although this hypothesis still requires experimental validation in plants.^5^ Consistent with this hypothesis, we show that AtGRP2 forms biomolecular condensates potentially mediated by cation−π interactions between Arg and Tyr residues and that these condensates are sensitive to pH and RNA, features that are often associated with stress-responsive regulation. Together, these results support the view that AtGRP2 can operate reversibly in dynamic cellular environments. Complementing these findings, previous work from our group showed that the AtGRP2 CSD adopts a conserved eukaryotic fold, with flexible loops and aromatic residues that are important for nucleic acid binding, including π interactions, the same interaction types implicated here as key drivers of PS.^25^ Moreover, the structural similarity to Lin28B, a miRNA-regulating protein, supports the functional hypothesis that AtGRP2 may be involved in post-transcriptional regulation in plants, possibly within Dicing bodies or the nucleolus. Collectively, our biochemical and structural data establish a mechanistic framework that explains the intrinsic phase separation behavior of AtGRP2 and provides a foundation for future *in vivo* studies aimed at elucidating its cellular functions in plant stress adaptation and RNA regulation.

## Methods

### Bioinformatic and Biostatistical Analysis

Bioinformatic analyses were performed using the AtGRP2 amino acid sequence retrieved from The *Arabidopsis* Information Resource (TAIR; AT4G38680). Disorder propensity was predicted using algorithms from the Predictor of Natural Disordered Regions (PONDR) family. PONDR was also used to generate charge–hydropathy analyses. Residue-level charge distribution and hydropathy profiles were obtained using the Classification of Intrinsically Disordered Ensemble Regions (CIDER) tool. The PScore for AtGRP2 was calculated using the Phase Separation Predictor (PSP) algorithm. Prion-like propensity was assessed using PLAAC (Prion-Like Amino Acid Composition). Granule-forming propensity was assessed using catGRANULE and compared with control proteins known to undergo or not undergo LLPS, based on amino acid sequences retrieved from UniProt. Statistical analyses were performed in GraphPad Prism (version 8.1.1) using analysis of variance (ANOVA), as indicated in the corresponding figure legends. Raw data preprocessing included checking for missing values, normalization when required, and identification of potential outliers; any outliers were handled according to predefined statistical criteria.

### Recombinant Protein Expression and Purification

Thio6His6-TEV-mEGFP-AtGRP2 and the corresponding mutant recombinant proteins were expressed in chemically competent *Escherichia coli* BL21­(DE3) cells (Supporting Table 1). After transformation with plasmid DNA and heat shock, cells were plated on LB agar supplemented with kanamycin and incubated overnight at 37 °C. A single colony was used to inoculate a 1 L culture, which was grown to an OD_600_ of ∼0.6 before protein expression was induced with 1 mM IPTG at 18 °C for 16 h. Cells were harvested by centrifugation and stored at −80 °C. For purification, cell pellets were resuspended in lysis buffer (50 mM Tris-HCl, pH 8.0, 500 mM NaCl, 5 mM imidazole, 300 μM PMSF) and lysed by sonication (10 cycles of 30 s pulses at 40% power). The lysate was cleared by centrifugation (8077g, 40 min, 4 °C), filtered (0.45 μm), and applied to a HisTrap HP Ni^2+^-affinity column. Bound proteins were eluted using a linear imidazole gradient (5–500 mM), and fractions were analyzed by SDS-PAGE. The eluted protein was treated with His_6_-TEV protease (5:1 substrate:protease ratio) and RNase A (0.2 mg/mL) for 24 h at room temperature during dialysis to remove the His_6_ tag and degrade bacterial RNA. All constructs generated for this study contained an N-terminal Thio6His6-TEV-mEGFP tag fused to AtGRP2 variants, and apparent molecular weights were verified by SDS-PAGE (Supporting Table 1). The sample was then reapplied to the Ni^2+^-affinity column, allowing the cleaved protein to flow through while residual contaminants remained bound. Final purification was achieved by size-exclusion chromatography on a Superdex 75 column in buffer containing 20 mM Tris-HCl (pH 7.5), 50 mM NaCl, and 200 μM ZnSO_4_. Protein purity and concentration were assessed by SDS-PAGE and the Pierce 660 nm protein assay, respectively. Samples were concentrated to 100 μM using 3 kDa MWCO centrifugal filters and stored at −80 °C. RNA removal was verified by RNA-PAGE (100 V, 1 h, TBE buffer) followed by GelRed staining. Gel electrophoresis was performed using the Bio-Rad Mini-PROTEAN system with 4.8% stacking and 12.5% resolving gels (Supporting Figure 4).

### Phase Separation Conditions

LLPS of mEGFP–AtGRP2 (10–20 μM) was investigated in buffer D (20 mM Tris-HCl, pH 7.5, 50 mM NaCl, 20–50 μM ZnSO_4_). Molecular crowding was tested using PEG 4000, PEG 20000, and Ficoll (1.0–12.5% (w/v)). Physicochemical drivers were probed using NaCl (50–1000 mM), arginine (50–200 mM), 1,6-hexanediol (1–25% (v/v)), and a pH range of 4.0–9.5. RNA effects were examined using total RNA extracts from *A. thaliana* (1–200 ng/μL) or a synthetic, rhodamine-labeled Lin28 RNA ligand (prelet-7g; 33 nt; 5′-/5hoR-XN/rUrGrA rGrGrG rUrCrU rArUrG rArUrA rCrCrA rCrCrC rGrGrU rArCrA rGrGrA rGrArU-3′) obtained from Integrated DNA Technologies (IDT) and used at 0.5–20 μM. dNTPs were used as controls at the same concentrations.

### LLPS Studies by Microscopy, Turbidity, and Saturation Concentration

Condensate formation was analyzed by phase-contrast and fluorescence microscopy in PEG-silanized chambers. Samples (10 μL) were imaged after 30 min incubation at room temperature using an EVOS M5000 microscope (40× phase-contrast and 60× fluorescence) or a Zeiss Elyra PS.1 confocal microscope (100× DIC). Fluorescence was recorded using 470/550 nm excitation/emission settings. Condensate number, size, and circularity were quantified from five 100 μm^2^ fields of view per condition using Fiji. Turbidity was assessed by measuring absorbance at 600 nm at room temperature using an ultramicro quartz cuvette (10 mm path length; 50 μL volume). Absorbance readings were collected in triplicate using an Evolution 60S spectrophotometer (Thermo Scientific). Blank-corrected values were obtained by subtracting the absorbance of protein-free samples. To estimate the concentration of the dilute phase, samples were centrifuged to sediment the condensed phase, and the supernatant was carefully collected for analysis. The saturation concentration was then determined by measuring absorbance at 475 nm at room temperature in a 96-well plate (150 μL per well). Samples were prepared in triplicate. Final concentrations were calculated as the mean of triplicate measurements after subtraction of the mEGFP background signal. Data analysis was performed using GraphPad Prism (version 8.1.1).

### Fluorescence Recovery after Photobleaching (FRAP)

FRAP experiments were performed using a 405 nm laser (500 cycles) on 5 μM mEGFP–AtGRP2 in the absence or presence of prelet-7g RNA (protein/RNA = 40:1) in 10% PEG. Under these conditions, condensates were small (∼1–2 μm in diameter), and the bleached area corresponded to a circular region of interest (ROI) with a diameter of ∼0.5–1 μm. Recovery was monitored for 75 s (10 technical replicates) and normalized using the equation (*F*_*∞*_ – *F*_0_)/(*F*_*i*_ – *F*_0_), where *F*_∞_ is the fluorescence intensity at the recovery plateau, *F*_0_ is the intensity immediately after photobleaching, and *F*_*i*_ is the prebleach fluorescence intensity. Data are presented as mean ± SD from *n* = 3 independent experiments (same purified protein batch). Statistical comparisons were performed using one-way ANOVA followed by Tukey’s multiple-comparisons test; *p* < 0.05 was considered statistically significant.

## Supplementary Material





## Notes

Description of the AtGRP2 constructs (Supporting Table 1); RNA binds AtGRP2 (Supporting Figure 1); phase separation of AtGRP2 constructs (Supporting Figure 2); phase separation of AtGRP2 constructs lacking Tyr residues and the N-terminal domain (Supporting Figure 3); purification of mEGFP-AtGRP2 (Supporting Figure 4) (PDF)
AtGRP2 forms gel-like condensates that assemble into clusters over time, representative time-course video showing AtGRP2 cluster formation over 1 h (Supporting Video 1) (MOV)

## ⊥

In posthumous memory of this committed, brilliant, passionate, visionary and enthusiastic young Brazilian scientific leader.
