# Supplementary material for: In Vitro Phase Separation Characterization of the Arabidopsis thaliana Glycine-Rich RNA-Binding Protein AtGRP2
Source: ACS Omega. 2026 May 21;11(22):32981–95. doi: 10.1021/acsomega.6c02622 (PMC13261584; doi:10.1021/acsomega.6c02622)
Supplement: Supplementary file 1 [file ao6c02622_si_001.pdf]

Running title: Phase separation of AtGRP2 in *Arabidopsis thaliana*

In vitro phase separation characterization of the *Arabidopsis thaliana* glycine-rich RNA-binding protein AtGRP2

Giovanna S. Melo[1], Gilberto Sachetto-Martins[2], André L.S. Santos[1,3*], Yraima Cordeiro[4*], Anderson S. Pinheiro[1#]

[1] Graduate Program in Biochemistry (PPGBq), Institute of Chemistry, Federal University of Rio de Janeiro, Rio de Janeiro 21941-909, Brazil

[2] Department of Genetics, Institute of Biology, Federal University of Rio de Janeiro, Rio de Janeiro 21941-902, Brazil

[3] Department of General Microbiology, Institute of Microbiology Paulo de Góes, Health Science Center, Federal University of Rio de Janeiro, Rio de Janeiro 21941-902, Brazil

[4] Faculty of Pharmacy, Federal University of Rio de Janeiro, Rio de Janeiro 21941-902, Brazil

*To whom correspondence should be addressed: andre@micro.ufrj.br (André Santos), yraima@farmacia.ufrj.br (Yraima Cordeiro)

[#]In posthumous memory of this committed, brilliant, passionate, visionary and enthusiastic young Brazilian scientific leader.

$Thio_6His_6$-TEV-mEGFP-AtGRP2	kDa	pI
Wild type ($AtGRP2_{1\text{-}203}$) 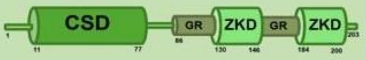	48.6	5.95
Δ C-terminal ($AtGRP2_{1\text{-}79}$) 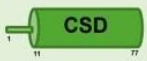	37.2	5.62
Δ N-terminal ($AtGRP2_{80\text{-}203}$) 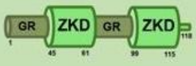	39.6	6.52
Δ Zinc finger ($AtGRP2_{1\text{-}169}$) 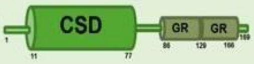	45.4	6.10
Δ Glycine ($AtGRP2_{1\text{-}122}$) 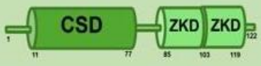	42.4	5.53
Δ Arginine ($AtGRP2_{1\text{-}189}$) 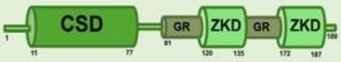	46.7	5.06
Δ Tyrosine ($AtGRP2_{1\text{-}195}$) 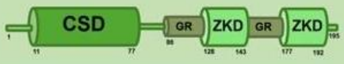	47.1	5.95

Supporting Table 1. AtGRP2 constructs scheme. Molecular weight (kDa) and isoelectric point (pI) of each construct are informed.

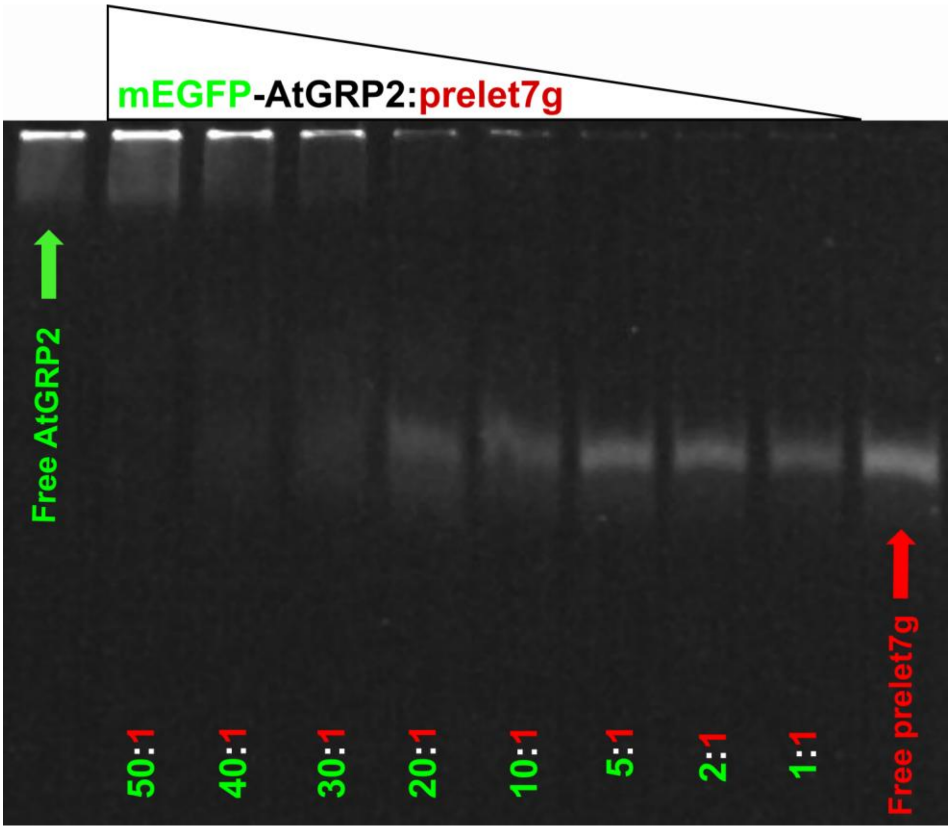

Supporting Figure 1. RNA binds AtGRP2. Electrophoretic Mobility Shift Assay (EMSA) shows that RNA is of decreasing protein:RNA molar ratios (50:1 to 1:1) shown below each lane at a fixed concentration of pre-let-7g (50 ng/mL, ~ 5 μM). Free AtGRP2 and free RNA are indicated in the first and last lanes, respectively.

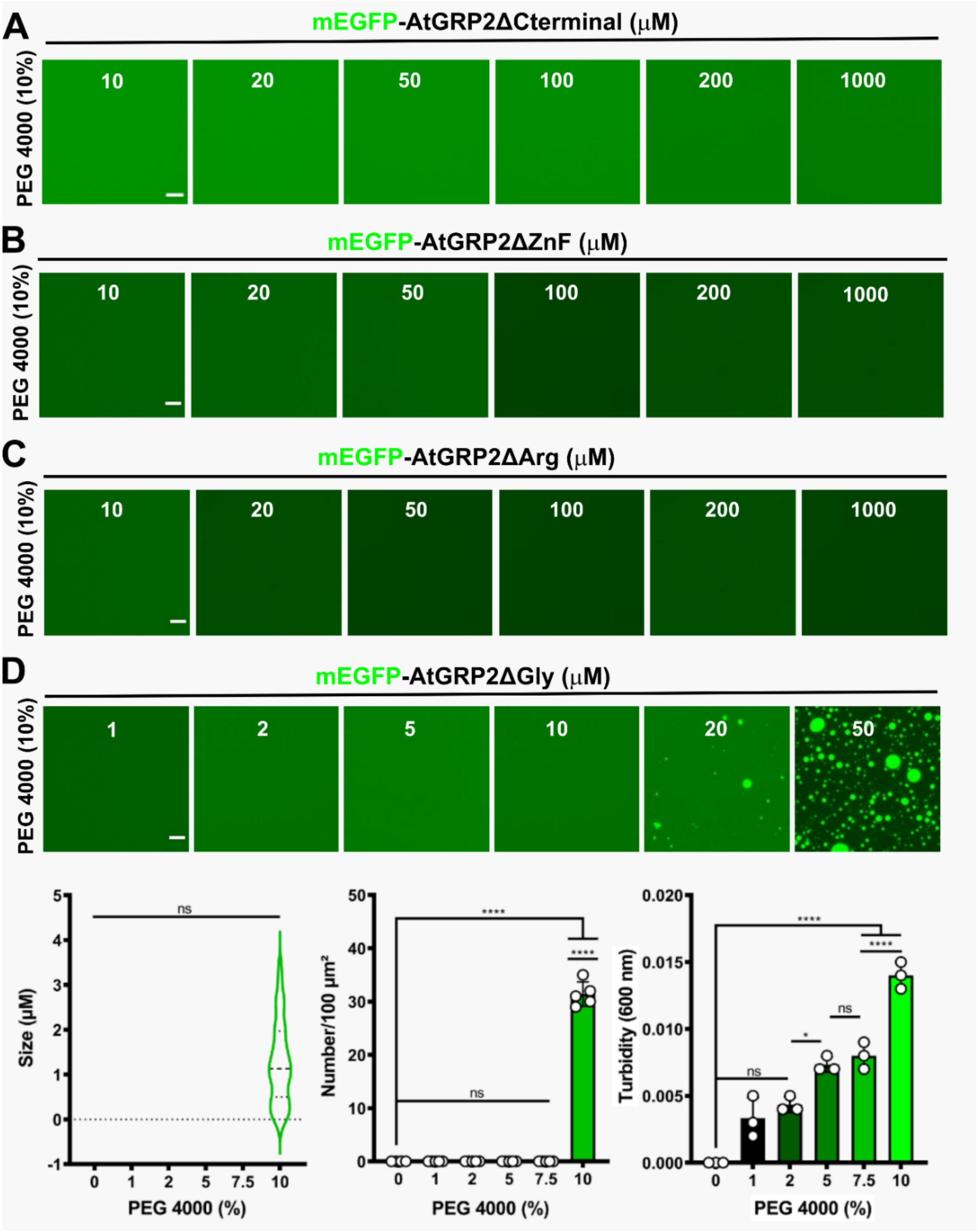

Supporting Figure 2. Phase separation of AtGRP2 constructs. Fluorescence microscopy showing that the isolated N-terminal CSD (AtGRP2ΔCterminal) (**A**), the construct lacking the zinc finger domains (AtGRP2ΔZnF) (**B**), and the arginine-deleted construct (AtGRP2ΔArg) (**C**) do not form condensates up to 1 mM. (**D**) Fluorescence microscopy and quantitative analysis (condensate number and size, together with turbidity measurements for AtGRP2ΔGly at 20 μM) showing that the construct lacking the glycine-rich region (AtGRP2ΔGly) undergoes condensation starting at 20 μM. Scale bars: 10 μm. Data in **D** (lower panels) are presented as mean ± SD; n = 3 independent experiments (same protein batch). Significance levels: ns ($p \geq 0.05$), *($p < 0.05$), **($p < 0.01$), ***($p < 0.001$), ****($p < 0.0001$).

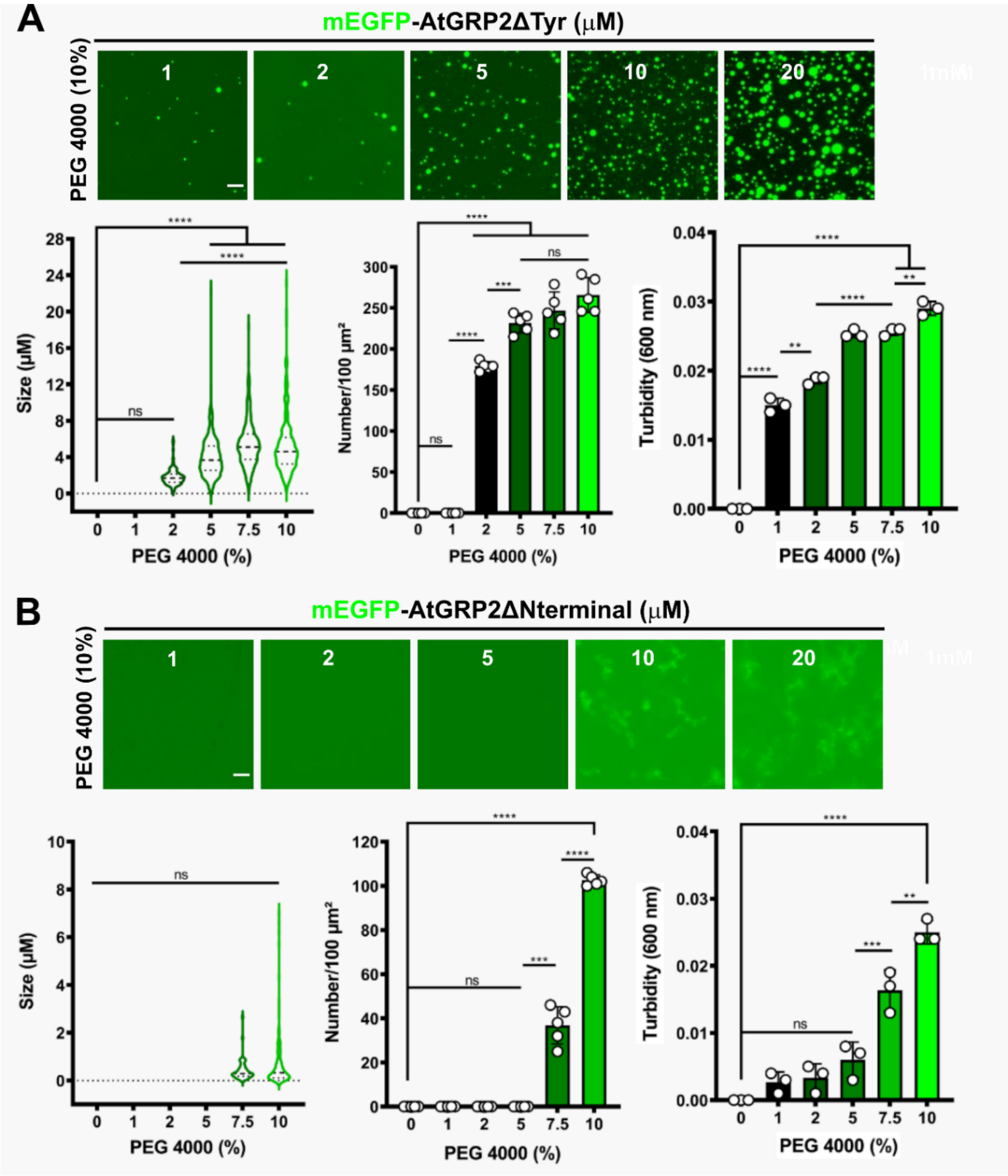

Supporting Figure 3. Phase separation of AtGRP2 constructs lacking Tyr residues and the N-terminal domain. (**A**) Fluorescence microscopy and quantitative analysis (condensate size and number, together with turbidity measurements for AtGRP2ΔTyr at 20 µM upon increasing PEG concentration) showing that the AtGRP2ΔTyr variant undergoes phase separation and displays an increased phase-separation propensity relative to full-length AtGRP2. (**B**) Fluorescence microscopy and quantitative analysis (condensate size and number, together with turbidity measurements for AtGRP2ΔNterminal at 20 µM upon increasing PEG concentration) showing that the isolated C-terminal region branched, solid-like assemblies rather than spherical condensates. Scale bars: 10 µm. Data in **A** and **B** (lower panels) are presented as mean ± SD; n = 3 independent experiments (same protein batch). Significance levels: ns ($p \geq 0.05$), *($p < 0.05$), **($p < 0.01$), ***($p < 0.001$), ****($p < 0.0001$).

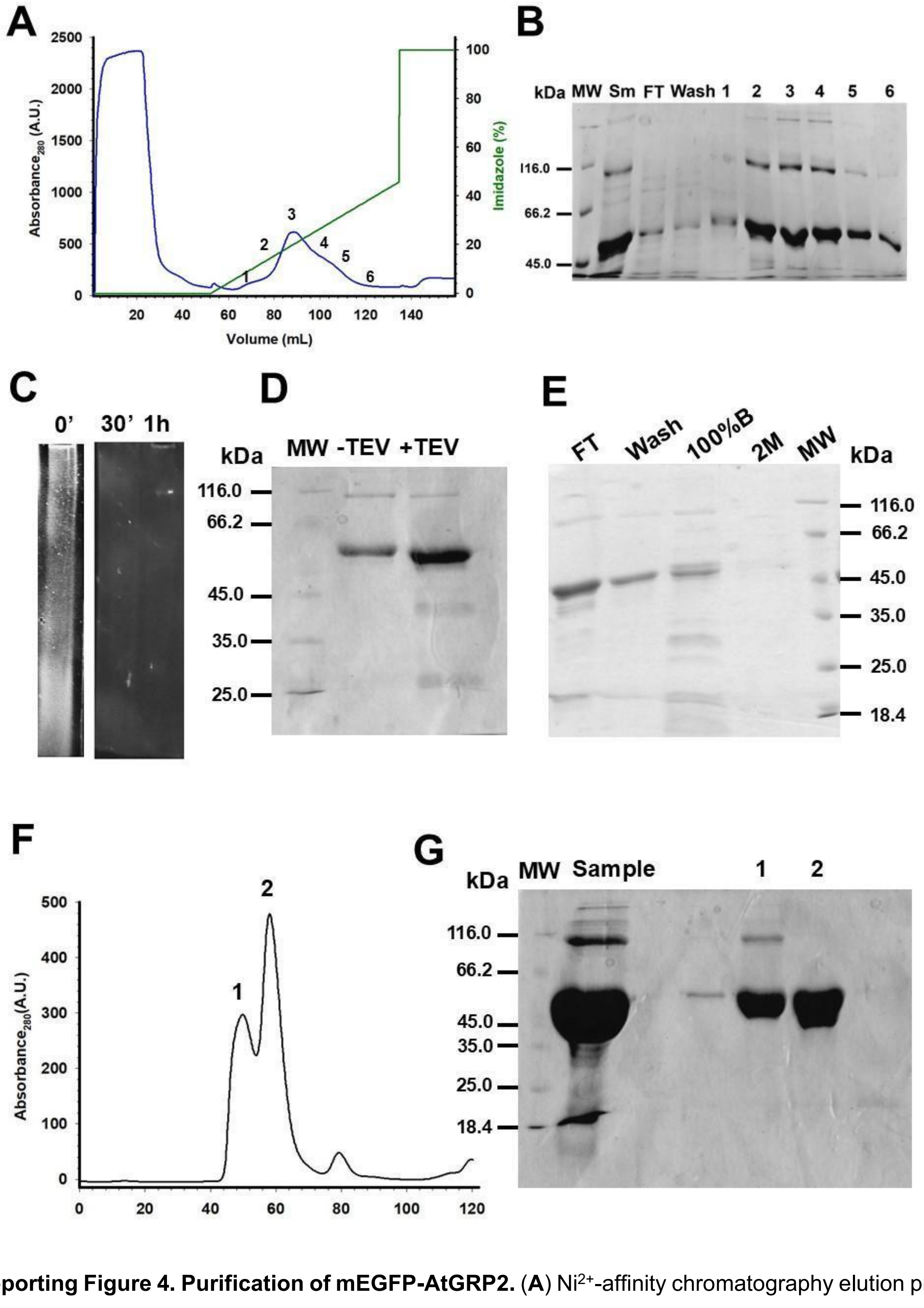

Supporting Figure 4. Purification of mEGFP-AtGRP2. (**A**) Ni^{2+}-affinity chromatography elution profile of the Thio6His6-TEV-mEGFP-AtGRP2 fusion protein. (**B**) SDS-PAGE analysis of elution fractions shown in **A**. (**C**) Agarose gel at 1% prepared in 1× TBE buffer and stained with GelRed® (1:1000) showing removal of bacterial RNA after RNase A treatment (0.2 mg/mL) for 30' and 1 hour. (**D**) SDS-PAGE (12%) analysis of His$_6$-tag cleavage by TEV protease. (**E**) SDS-PAGE analysis of TEV-cleaved samples after Ni^{2+}-affinity chromatography showing that mEGFP–AtGRP2 is recovered in the flow-through (FT)/wash fractions. (**F**) Size-exclusion chromatography (SEC) profile of mEGFP–AtGRP2. (**G**) 12% SDS-PAGE analysis confirming sample purity after SEC (fraction 2 from the chromatogram shown in **F**).
